# Population pharmacokinetics of cabotegravir following intramuscular thigh injections in adults with and without HIV

**DOI:** 10.1128/aac.00880-24

**Published:** 2024-10-23

**Authors:** Kelong Han, Ronald D. D'Amico, William R. Spreen, Susan L. Ford

**Affiliations:** 1 GSK, Collegeville, Pennsylvania, USA; 2 ViiV Healthcare, Durham, North Carolina, USA; 3 GSK, Durham, North Carolina, USA; Providence Portland Medical Center, Portland, Oregon, USA

**Keywords:** cabotegravir, intramuscular thigh injection, HIV, long-acting, population pharmacokinetics

## Abstract

Cabotegravir intramuscular gluteal injection is approved for HIV treatment (with rilpivirine) and prevention. Thigh muscle is a potential alternative injection site. We aim to characterize cabotegravir pharmacokinetics and its association with demographics following intramuscular thigh injection in comparison with gluteal injection using population pharmacokinetic (PPK) analysis. Fourteen HIV-negative participants received 600 mg single thigh injection in phase 1 study 208832 and 118 participants with HIV received thigh injections 400 mg monthly 4× or 600 mg once-every-2-months 2× after ≥3 years of gluteal injections in phase 3b study ATLAS-2M provided 1,249 cabotegravir concentrations from 366 thigh injections and 1,998 concentrations from 1,618 gluteal injections. The established gluteal PPK model was modified by adding thigh injection compartment and fit to pharmacokinetic data following both gluteal and thigh injections, enabling within-person comparison in ATLAS-2M. Gluteal parameters were fixed. Similar to the gluteal absorption rate constant (KA_gluteal_), the thigh absorption rate constant (KA_thigh_) was slower in females than males and in participants with higher BMI. KA_thigh_ was strongly correlated with KA_gluteal_ (correlation coefficient 0.766), best described by the additive linear relationship KA_thigh_ = KA_gluteal_ + 0.0002527 h^−1^. Terminal half-life of thigh injection was 26% (male) and 39% (female) shorter than gluteal injection. Relative bioavailability of thigh to gluteal was estimated to be 89.9%. The impact of covariates on cabotegravir exposure following thigh injections was ≤35%. In conclusion, cabotegravir absorption following thigh injection was correlated with, faster than, and 10% less bioavailable than gluteal injection, and correlated with sex and BMI. The cabotegravir thigh PPK model can inform dosing strategies and future study design.

## INTRODUCTION

Cabotegravir (CAB) is a small-molecule human immunodeficiency virus (HIV) integrase strand transfer inhibitor (INSTI) (1–6). Long-acting (LA) CAB plus LA rilpivirine (RPV) administered monthly (QM) or once every 2 months (Q2M) via intramuscular (IM) ventrogluteal (recommended injection site) (7) or dorsogluteal injections is the first and only complete LA regimen approved for maintenance of HIV-1 virologic suppression in people living with HIV (https://www.eacsociety.org/media/final2021eacsguidelinesv11.0_oct2021.pdf; https://clinicalinfo.hiv.gov/en/guidelines/hiv-clinical-guidelines-adult-and-adolescent-arv/whats-new-guidelines; 8, 9). Phase 3 studies in people living with HIV demonstrated that the QM regimen of CAB LA + RPV LA was non-inferior to standard oral therapy in maintaining HIV-1 suppression (10, 11), and the Q2M regimen was non-inferior to the QM regimen (12). CAB LA single-agent administered Q2M via IM gluteal injection is the first and only LA injectable approved for HIV-1 pre-exposure prophylaxis (PrEP) after demonstrating superiority to the standard-of-care of daily oral tenofovir disoproxil fumarate/emtricitabine in phase 3 studies (13–15).

CAB LA exhibits absorption-limited (flip-flop) kinetics (the absorption rate is slower than the elimination rate) as the terminal half-life (*T*1/2) of 25–54 days following IM gluteal injection was substantially longer than the *T*1/2 of 35–42 h following oral dosing (3, 4). The CAB population pharmacokinetic (PK) model for oral tablet and IM gluteal injection (oral + gluteal model) was established based on 23,926 plasma concentrations from 1,647 adults with HIV (72%) and without HIV (28%) in 16 clinical studies (16). The oral + gluteal model was a two-compartment model with first-order absorption and elimination for both routes of administration (16). Clearances and volumes were scaled to body weight. The estimated relative bioavailability of oral to LA was 75.6% (16). Race and age were not significant covariates. The absorption rate constant for the LA IM gluteal injection (KA_gluteal_) was 50.9% lower in females and 47.8% higher if the IM gluteal injection was given as two split injections (16). KA_gluteal_ decreased with increasing body mass index (BMI) and decreasing needle length (16). Clearance was 17.4% higher in current smokers (16). The impact of any covariate was ≤32% on CAB trough and peak concentrations following QM and Q2M regimens of IM gluteal injections. The final oral + gluteal model was validated by adequately predicting 5,097 plasma concentrations from 647 participants who were not included in the model-building dataset (16).

The *vastus lateralis* (lateral thigh) muscle is a potential alternative site for IM injections in cases of fatigue or intolerability of the gluteal injection site, inaccessibility of the gluteal muscle (e.g., buttock implants or insufficient gluteal mass), or physical inconvenience (e.g., prior to prolonged sitting). The lateral thigh muscle has been used as an injection site for other drugs and vaccines and is commonly utilized in children (https://www.cdc.gov/vaccines/hcp/admin/downloads/IM-Injection-Infants-508.pdf) (17). Two clinical studies have been conducted to evaluate CAB IM thigh injections. The phase 1 study 208832 of single-dose CAB + RPV LA IM thigh injections in 14 participants without HIV supported the further evaluation of IM thigh injections (18). The thigh PK substudy of the ongoing phase 3b ATLAS-2M study evaluated short-term repeat IM thigh injections of CAB + RPV LA in 118 people living with HIV who received ≥3 years of IM gluteal injections (19). Data from both studies suggested potentially faster absorption of CAB via IM thigh injection than gluteal injection and warranted further quantitative analysis and comparison of PK.

The objectives of this analysis were to quantitatively characterize CAB PK and its association with intrinsic and extrinsic factors following CAB IM thigh injections in comparison with gluteal injections in adult participants both without and with HIV using population PK modeling by combining the data from the phase 1 study 208832 and the phase 3b ATLAS-2M substudy. The CAB population PK model for IM thigh injections can be used for PK simulations to inform dosing strategies and future study design.

## RESULTS

### Model-building data set

**Fig 1:**
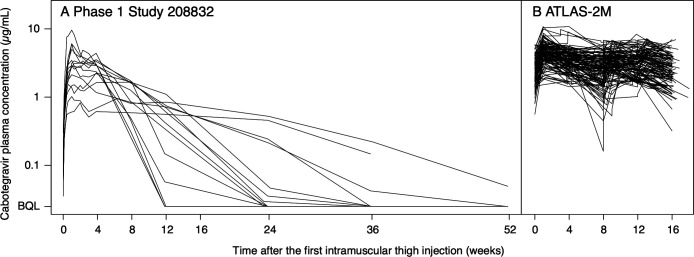
Individual concentration-versus-time profiles in the model-building data set from (**A**) study 208832 and (**B**) ATLAS-2M thigh PK substudy. BQL, below the quantification limit of 0.025 µg/mL.

A total of 3,481 CAB plasma concentrations from 132 adult participants were included in the model-building data set: 1,249 concentrations following 366 thigh injections, 1,998 concentrations following 1,618 gluteal injections, and 234 concentrations following oral administration. CAB doses administered as thigh injections were 400 and 600 mg. Individual concentration-versus-time profiles following thigh injections are displayed in Fig. 1. Demographics and key clinical variables are summarized in Table 1.

**TABLE 1:** Summary of baseline characteristics of participants included in the analysis

Characteristic	Value	
Number of participants, *n*	132	
Number of concentrations, *n*	3,481	
Age, median (range), years	42.5 (20–67)	
Body weight, median (range), kg	75.75 (50.1–129.7)	
Body mass index, median (range), (kg/m^2^)	25.35 (17.87–50.9)	
Female (assigned sex at birth), *n* (%)	50 (37.88%)	
People living with HIV, *n* (%)	118 (89.39%)	
Race, *n* (%)		
White	104 (78.79%)	
Black or African American	24 (18.18%)	
Asian	2 (1.52%)	
Other races	2 (1.52%)	
Smoking status, *n* (%)		
Never smoked	75 (56.8%)	
Former smoker	21 (15.9%)	
Current smoker	36 (27.3%)	

### Population PK modeling

**Fig 2:**
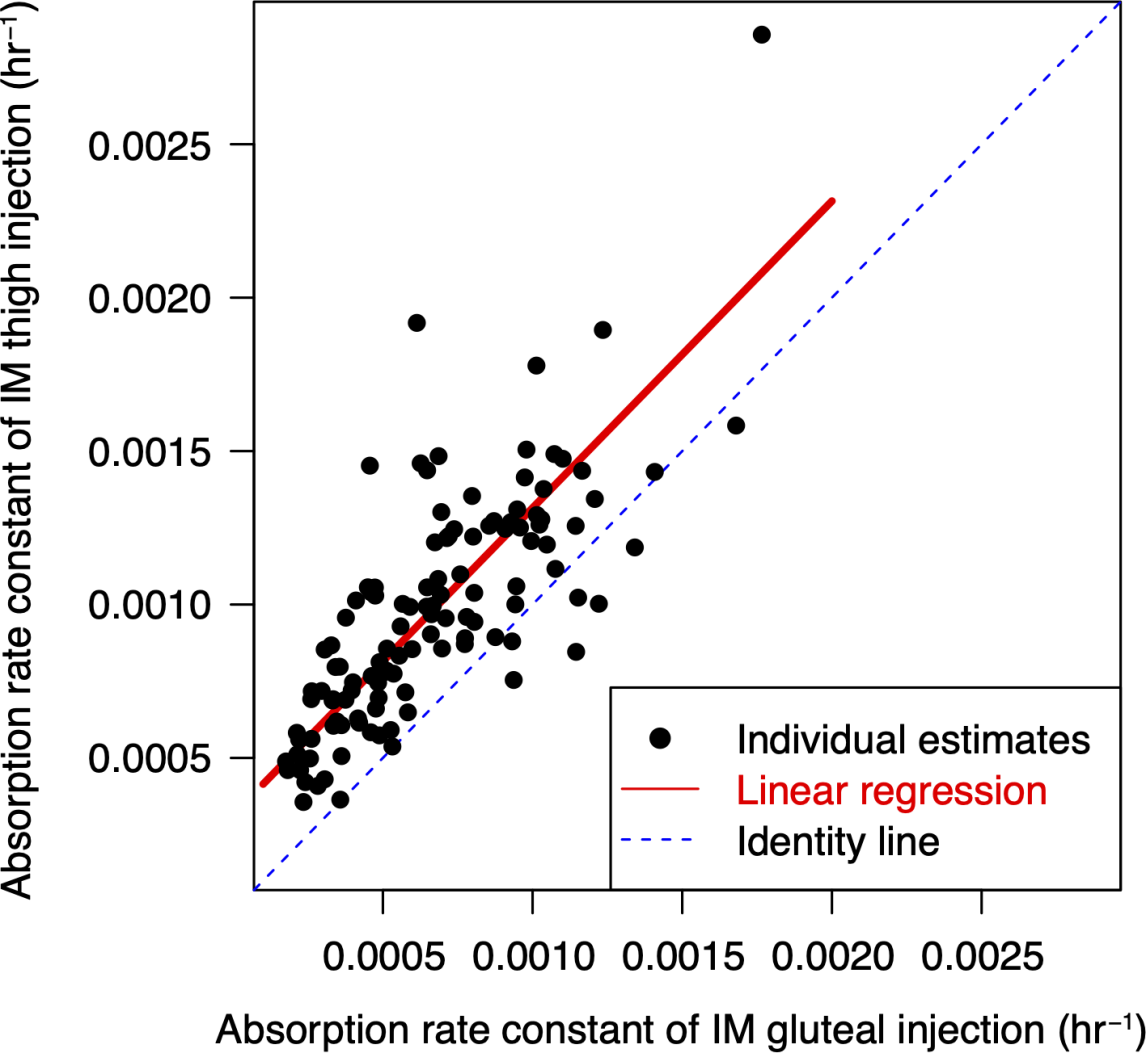
Intra-individual correlation of post-hoc estimates of absorption rate constants for gluteal and thigh injections in the ATLAS-2M thigh PK substudy. IM, intramuscular.

Post-hoc estimates of KA_gluteal_ and the absorption rate constant for the LA IM thigh injection (KA_thigh_) were correlated with a Pearson correlation coefficient of 0.766 in the 118 participants who received both gluteal and thigh injections of CAB in the ATLAS-2M thigh PK substudy (Fig. 2), and therefore, the correlation was deemed strong.

**Fig 3:**
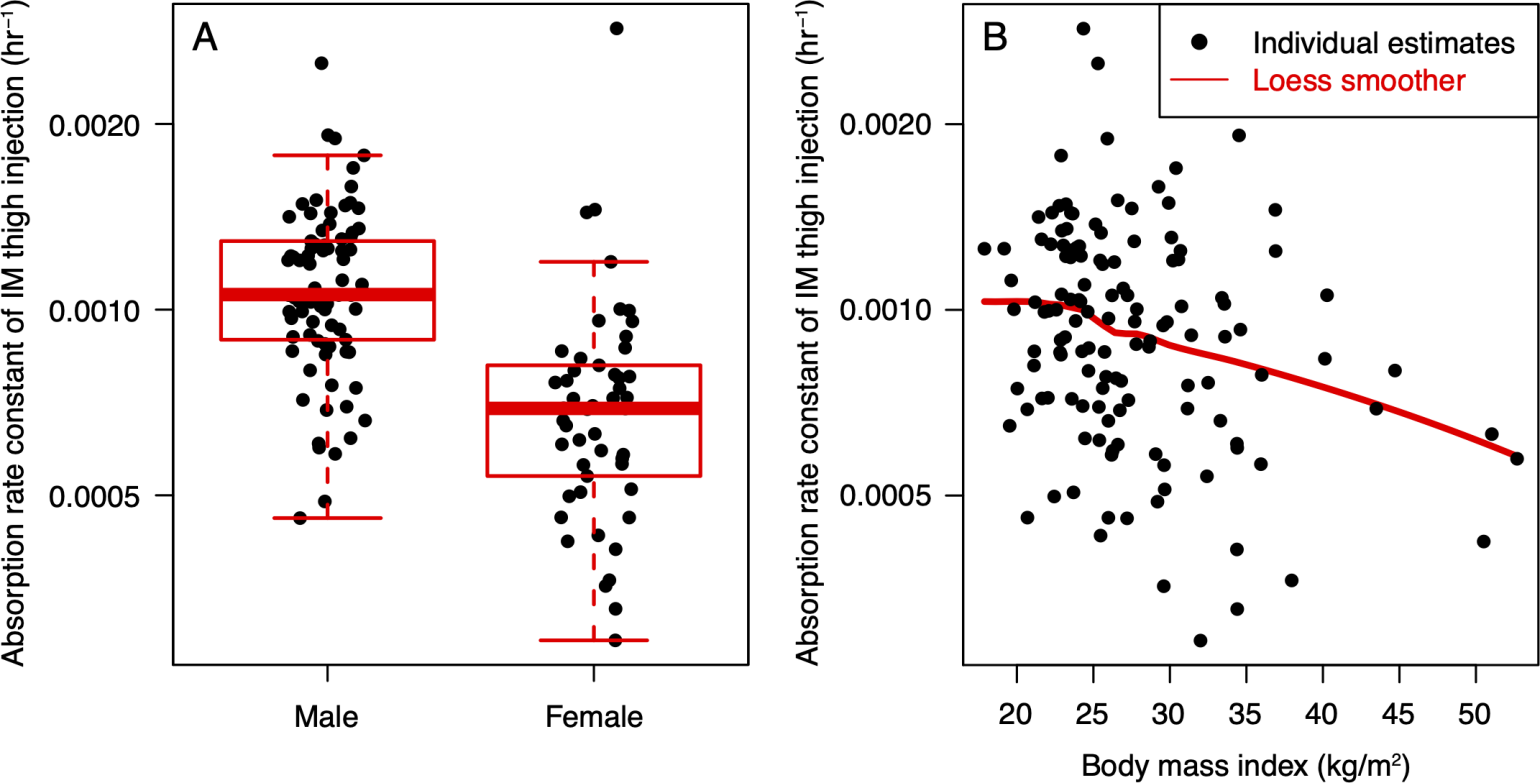
Correlation of the absorption rate constant of intramuscular thigh injection with (**A**) sex at birth and (**B**) body mass index. IM, intramuscular.

KA_thigh_ was lower in females than males by 34.5% (Fig. 3A), decreasing with increasing BMI (Fig. 3B) for both sexes, and was similar between both studies. Objective function value (OFV) decreased by 34 when the sex effect on KA_thigh_ was estimated (*P* < 5 × 10^−9^), and decreased by 11.4 when the effect of BMI on KA_thigh_ was estimated (*P* < 0.0005), further confirming the statistically significant association of KA_thigh_ with sex and BMI. KA_thigh_ was not associated with HIV status. Relative bioavailability of the thigh injection relative to the gluteal injection (F_thigh/gluteal_) was not associated with any covariate evaluated and not associated with KA_gluteal_ or KA_thigh_, consistent with the previous observation that the relative bioavailability of the oral tablet relative to the gluteal injection (F_oral/gluteal_) was not associated with any covariate evaluated.

Given that (i) a strong correlation was observed between KA_gluteal_ and KA_thigh_ and (ii) the covariate relationships on KA_thigh_ were deemed similar to those on KA_gluteal_, KA_thigh_ was modeled as a function of KA_gluteal_. Various linear functions and power functions were evaluated to model KA_thigh_ as a function of KA_gluteal_ (Supplemental Table). Regardless of the assumption of the underlying distribution of the random effect (lognormal or normal distributions), directly assessing inter-individual variability (IIV) on KA_thigh_ provided better fitting than assessing IIV on the difference between KA_thigh_ and KA_gluteal_. OFV decreased by 6–12.8 points when IIV was assessed directly on KA_thigh_ instead of being assessed on the difference between KA_thigh_ and KA_gluteal_. A linear function with both proportional and additive components (i.e., KA_thigh_ = *a* * KA_gluteal_ + *b*, where *a* and *b* were estimated) and a power function (i.e., KA_thigh_ = *a* * KA_gluteal_^*b*^, where *a* and *b* were estimated) both led to slightly lower OFV than a simple linear function with the additive component only (i.e., KA_thigh_ = KA_gluteal_ + *c*, where *c* was estimated), but the reduction in OFV was 2.8 and 3, respectively, which did not meet the pre-specified statistical criterion of 3.84 for 1 df (*P* < 0.05). Therefore, KA_thigh_ = KA_gluteal_ + 0.0002527 h^−1^ was selected as the final model.

PK parameter estimates of the final model are summarized in Table 2 and displayed in the equation below:


$$KA_{thigh}=0.0002527+\;0.00073\times \left(1-50.9\%\;if\;female\right)\times \left(1+47.8\%\;if\;split\right)\times {\left(\frac{BMI}{25.4}\right)}^{-0.766}\times \;{\left(\frac{NDL}{1.5}\right)}^{0.478}$$

where “split” indicates that the LA IM injection was given as two split injections, and NDL denotes needle length in inches.

**TABLE 2:** Parameter estimates of the final model^*a*^

Parameter	Estimate [90% CI from bootstrap]	RSE (%)	IIV [RSE] (%)	Shrinkage (%)
KA_thigh_ − KA_gluteal_ (h^−1^)	0.0002527 [0.0001559, 0.0003573]	20.7		
KA_thigh_ (h^−1^)			41.6 [9.2]	24.6
*T*1/2 male (weeks)	4.2			
*T*1/2 female (weeks)	6.4			
*F*_thigh/gluteal_	89.9% [85.7%, 94.7%]	2.9	22.9 [10.8]	27.5
KA_gluteal_ (h^−1^)	0.000733 (fixed)			
CL/F (L/h)	0.151 (fixed)			
V2/F (L)	5.27 (fixed)			
Q/F (L/h)	0.507 (fixed)			
V3/F (L)	2.43 (fixed)			
*F*_oral/gluteal_	75.6% (fixed)			
Female vs male on KA_gluteal_	−50.9% (fixed)			
BMI exponent on KA_gluteal_	−0.766 (fixed)			
Split injection on KA_gluteal_	47.8% (fixed)			
NDL exponent on KA_gluteal_	0.478 (fixed)			

^*a*^ BMI, body mass index (kg/m^2^); CI, confidence interval; CL/F, apparent central clearance; Fixed, PK parameters not related to thigh injection were fixed to the values from the final model for oral tablet and gluteal injection (16); *F*_oral/gluteal_, relative bioavailability of oral tablet relative to gluteal injection; *F*_thigh/gluteal_, relative bioavailability of thigh injection relative to gluteal injection; IIV, inter-individual variability; IM, intramuscular; KA_gluteal_, absorption rate constant for the LA IM gluteal injection; KA_thigh_, absorption rate constant for the LA IM thigh injection; LA, long-acting; NDL, needle length (inch); Q/F, apparent intercompartmental clearance; RSE, relative standard error; *T*1/2, terminal half-life; V2/F, apparent central compartment volume of distribution; V3/F, apparent peripheral compartment volume of distribution.

Bioavailability of the thigh injection was estimated to be 89.9% that of the gluteal injection (*F*_thigh/gluteal_). All PK parameters were estimated with good precision, as evidenced by the relative standard error of <21%. Based on these estimates, the CAB *T*1/2 following IM thigh injections was calculated to be 4.2 weeks in males and 6.4 weeks in females, who had the population median BMI of 25.4 kg/m^2^ and received unsplit thigh injections using a 1.5-inch needle. These CAB T1/2 values following IM thigh injections were 25.8% shorter than the *T*1/2 values following gluteal injections in males (4.2 vs 5.7 weeks) and 39.3% shorter in females (6.4 vs. 10.6 weeks).

**Fig 4:**
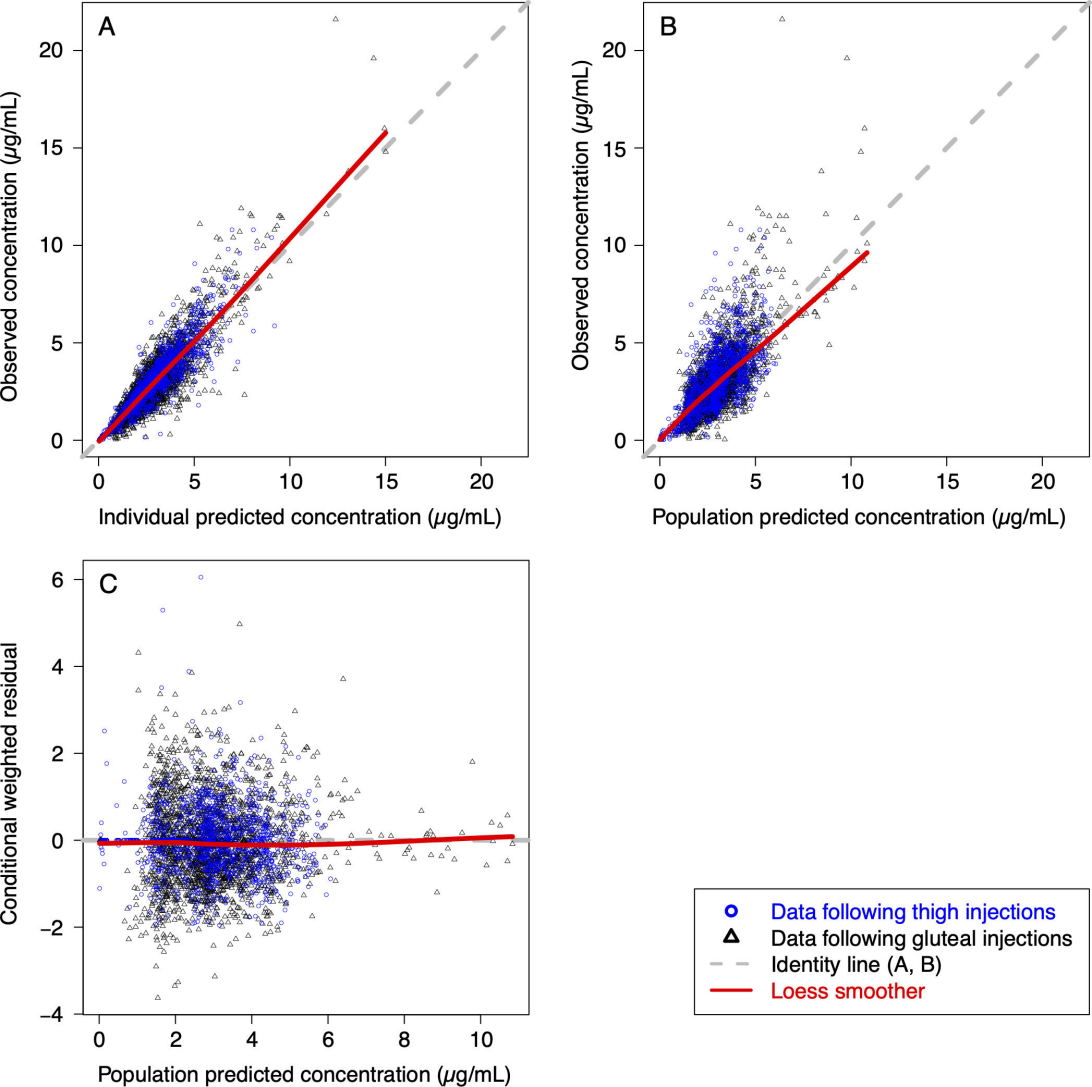
Goodness-of-fit plots of the final model.

The goodness-of-fit plots from the final model (Fig. 4) demonstrated good agreement between predicted and observed concentrations, with no apparent bias in residual. Bootstrapping (Table 2) resulted in median parameter estimates and 90% confidence intervals (5th and 95th percentiles of the bootstrap replicates) similar to the estimates from the model-building data set. Based on prediction-corrected visual predictive check (pcVPC, Supplemental Figure), the model predictions adequately captured the prediction-corrected concentration-time profiles within the 90% prediction interval (5th and 95th percentiles).

**Fig 5:**
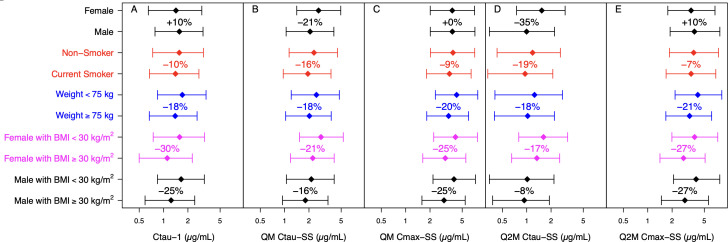
Relative impact of covariates on cabotegravir plasma concentrations following thigh injections based on simulations: (**A**) *C*_tau_-1, (**B**) *C*_tau_-SS following QM regimen, (**C**) *C*_max_-SS following QM regimen, (**D**) *C*_tau_-SS following Q2M regimen, and (**E**) *C*_max_-SS following Q2M regimen. Each bar represents the 5th to 95th percentiles, with the median (diamond) in each subgroup within the covariate or covariate combination. Percentages above each bar represent the percent change in median vs the median of the reference subgroup within the same covariate or covariate combination (i.e., vs the bar of the same color above it as the reference). BMI, body mass index (kg/m^2^); *C*_max_, maximum concentration; *C*_tau_, trough concentration at the end of the dosing interval; *C*_tau_-1, *C*_tau_ following the first injection of 600 mg for both QM and Q2M injections; QM, once every month; Q2M, once every 2 months; SS, steady state (following the 11th injection of the QM injections or the 6th injection of the Q2M injections).

The relative impact of covariates retained in the final model on CAB exposure following IM thigh injections is demonstrated in Fig. 5. BMI had the greatest impact on CAB trough concentration (*C*_tau_) following the first injection (*C*_tau_-1) among all covariates or covariate combinations (Fig. 5A). Median *C*_tau_-1 was up to 30% lower in participants with BMI ≥30 kg/m^2^ than participants with BMI <30 kg/m^2^. Sex and smoking status led to a ≤ 10% change in *C*_tau_-1. Sex had the greatest impact on trough concentration at steady state (*C*_tau_-SS; Fig. 5B and D). Median *C*_tau_-SS was 21% (QM thigh injections) and 35% (Q2M thigh injections) lower in males than females. For QM thigh injections, the impact of all covariates was ≤21% on *C*_tau_-SS (Fig. 5B) and ≤25% on maximum concentration (*C*_max_) at steady state (*C*_max_-SS; Fig. 5C). For Q2M thigh injections, the impact of all covariates was ≤35% on *C*_tau_-SS (Fig. 5D) and ≤27% on *C*_max_-SS (Fig. 5E).

## DISCUSSION

This analysis is a population PK evaluation of CAB IM thigh injections in comparison with gluteal injections in adult participants both without and with HIV from phase 1 and 3 studies. The model-building data set included both single-dose and multiple-dose administrations, and both intensively and sparsely sampled CAB plasma concentration data. The CAB LA absorption rate following IM thigh injections was correlated with and generally faster than that of gluteal injections, slower in females than males, and slower in participants with higher BMI.

Information comparing PK between IM thigh and gluteal injections in humans is scarce in the public domain, and this analysis provides one of the first insights. In addition, there is limited information about the impact of intrinsic and extrinsic factors on PK of IM thigh injections, and this analysis is one of the first population PK models for IM thigh injections.

The correlation of the CAB LA absorption rate with sex and BMI that was observed for thigh injections was also observed for gluteal injections (16). The underlying mechanism for the impact of sex and BMI is likely similar for CAB thigh and gluteal injections and is potentially also the underlying mechanism for the faster CAB LA absorption via thigh injection than gluteal injection. Two mechanisms have been hypothesized: (i) higher vascularity leads to faster CAB LA absorption and (ii) CAB LA absorption is slower in subcutaneous (SC) fat than muscle, partially due to lower vascularity in SC fat.

Based on these possible mechanisms, faster CAB LA absorption via thigh injection is possibly caused by the higher vascularity in the thigh muscle than the gluteal muscle (20). In addition, it is well known that doses administered via IM injection are often partially injected into SC fat or have leaked into SC fat from muscle depot (17, 21–25). Due to the thinner SC fat layer in the thigh than the buttocks, the CAB IM dose is less likely to be SC, or a smaller portion of the CAB IM dose is SC, resulting in faster CAB LA absorption via thigh injection than gluteal injection. Similarly, the CAB IM dose is more likely to be SC, or a bigger portion of the CAB IM dose is SC, in females and participants with higher BMI (17, 21–25), resulting in slower CAB LA absorption.

The second possible mechanism mentioned above, that SC absorption is slower than IM absorption for CAB, is yet to be studied but has been observed with many other drugs (26–33). If the cause is the lower vascularity in SC fat than muscle, then for drugs with extremely slow IM/SC absorption, the IM absorption rate should be similar to the SC absorption rate because the vascularity is no longer the rate-limiting step for the absorption, and therefore, the IM absorption rate should not be associated with sex and BMI. This matches the observation with RPV LA (34) that the RPV IM absorption rate is five times slower than CAB and is not associated with sex or BMI.

The third possible mechanism mentioned above, that IM injection was often SC or partially SC, is supported by several prospective studies that radiologically determined if IM gluteal injections were truly IM (22–26). Collectively, these studies concluded that 48%–68% of IM injections were entirely or partially SC. Specifically for CAB, the phase 1 study 201767 (35) used magnetic resonance imaging (MRI) to assess CAB depot localization following ultrasonographic-guided IM gluteal injection and concluded that more injections were entirely or partially SC (*n* = 5) than truly IM (*N* = 2) despite the ultrasonographic guidance.

Due to the pre-specified criteria of (i) a strong correlation between KA_gluteal_ and KA_thigh_ and (ii) similar covariate relationships for KA_gluteal_ and KA_thigh_, KA_thigh_ was modeled as a function of KA_gluteal_. This approach is physiologically plausible because many of the known and unknown factors that affect CAB IM absorption are likely the same or similar within the same individual. In addition, this approach may also help avoid potential statistical caveats such as overparameterization for the size of the data set, low identifiability given the limited PK sampling schedule in the ATLAS-2M study, and inconclusive evaluation of covariate relationships due to an insufficient range of certain covariates.

Due to the fixed increase in the LA absorption rate constant associated with thigh dosing (KA_thigh_ = KA_gluteal_ + 0.0002527 h^−1^), individuals with slow gluteal absorption (participants with low KA_gluteal_) may experience a larger percentage increase in absorption rate than individuals with fast gluteal absorption (participants with high KA_gluteal_). The 5th and 95th percentiles of KA_gluteal_ post-hoc estimates in the model-building data set of the oral + gluteal model were 0.000215 h^−1^ and 0.001428 h^−1^. Participants with a low KA_gluteal_ of 0.000215 h^−1^ would experience a 118% increase in absorption rate (KA_thigh_ = 0.0004677 h^−1^) when switching from gluteal to thigh injections, whereas participants with a high KA_gluteal_ of 0.001428 h^−1^ would only experience an 18% increase (KA_thigh_ = 0.0016807 h^−1^).

To evaluate the relative impact of covariates on CAB exposure following IM thigh injections, individual PK parameters of each virtual participant were calculated from the population parameter estimates and participant-specific covariates of body weight, sex, BMI, and smoking status, which were sampled with replacement from the participants within this subgroup in the model-building data set. This maintained the correlations between covariates and the physiologically relevant spread of the other covariates (the inter-covariate variance relationships). Therefore, each subgroup reflected not only the effect of the covariate of choice but also the influence of any co-varying covariates. All injections were assigned with the most common needle length of 1.5 inches. The impact of sex, smoking status, body weight, and BMI on CAB *C*_tau_ and *C*_max_ concentrations following CAB IM thigh injections is similar to that following CAB IM gluteal injections.

One of the main applications of the CAB population PK model for IM thigh injections is to perform simulations under various scenarios that can inform dosing strategies and study design, especially considering that the clinical development of CAB remains active (36). This is particularly important for LA medications because the long *T*1/2 determined by slow LA absorption leads to a prolonged time to reach steady state and a prolonged time to achieve non-quantifiable concentrations upon discontinuation of dosing. This results in long durations of clinical studies and a long washout period, which may impact the study feasibility and may expose study participants unnecessarily. PK simulations using this model will be presented in future publications.

This analysis has several limitations. Some intrinsic and extrinsic factors that could affect PK following IM thigh injections could not be evaluated. For the IM gluteal injection, CAB LA absorption was faster when split injections or longer needle length were used, possibly due to greater surface area for absorption and penetration. Unfortunately, the data set in this analysis only included non-split injections, and only 3 out of the 366 thigh injections (0.8%) were administered using a needle length that was not 1.5 inches. Therefore, the impact of split injections and needle length on PK of CAB IM thigh injections could not be evaluated. In addition, the data set included no transgender participants, who are part of the target population for treatment and prevention of HIV-1.

In conclusion, CAB LA absorption following IM thigh injection was correlated with, and generally faster than, IM gluteal injection and is best described by the additive linear relationship KA_thigh_ = KA_gluteal_ + 0.0002527 h^−1^. CAB LA absorption following IM thigh injection was slower in females than males and slower in participants with higher BMI. The bioavailability of the thigh injection was estimated to be 89.9% that of the gluteal injection. The CAB population PK model developed for the IM thigh injection adequately characterized CAB PK and sources of variability in exposure to inform dosing strategies and future study design.

## MATERIALS AND METHODS

### Data

The study designs of the phase 1 study 208832 (NCT04371380) (18) and the thigh PK substudy of the phase 3b ATLAS-2M study (NCT03299049) (19) included in the analysis are compared in Table 3. In ATLAS-2M, only those participants who received at least one thigh injection were included in this analysis. All studies complied with the Declaration of Helsinki and Good Clinical Practice guidelines and were approved by the Institutional Review Board (IRB) of participating institutions. All study participants provided written informed consent.

**TABLE 3:** Summary of studies included in the analysis^*a*^

	Phase 1 study 208832	Thigh PK substudy of the phase 3b ATLAS-2M study
Participants	Adults without HIV	Adults with HIV
Prior CAB exposure	None	On CAB QM or Q2M regimen of IM gluteal injections for ≥3 years

Arm	Single thigh injection	QM thigh injection	Q2M thigh injection
Number of participants	14	64	54
Female, *n* (%)	5 (36%)	26 (41%)	19 (35%)
CAB dose of IM thigh injection	600 mg (3 mL)	400 mg (2 mL)	600 mg (3 mL)
CAB dosing regimen	4 weeks of daily oral CAB 30 mg, followed by a washout period of 10–14 days and a single 600 mg IM thigh injection	Final IM gluteal injection at Week −4, then 4 IM thigh injections of 400 mg on Day 1, at Weeks 4, 8, and 12, then resume IM gluteal injections at Week 16	Final IM gluteal injection at Week −4, then 2 IM thigh injections of 600 mg on Day 1 and at Week 8, then resume IM gluteal injections at Week 16

^*a*^ CAB, cabotegravir; IM, intramuscular; QM, once every month; Q2M, once every 2 months.

Human plasma samples were analyzed for CAB concentration using a validated analytical method (1–6) based on protein precipitation, followed by high-performance liquid chromatography with tandem mass spectrometry analysis, which has a 1,000-fold linear range with a lower limit of quantification (LLOQ) of 0.025 µg/mL and an upper limit of quantification of 25 µg/mL.

The covariates collected for evaluation included those related to demographics, laboratory tests, and injection-related factors including needle length and gauge. Lab tests included for evaluation as covariates were albumin, total bilirubin, direct bilirubin, alkaline phosphatase (ALP), alanine aminotransferase (ALT), aspartate aminotransferase (AST), gamma glutamyl transferase (GGT), lactate dehydrogenase (LDH), creatinine, creatinine clearance, urea, and HIV-1 viral load (log-transformed and non-transformed both as the continuous and the categorical variable). Both baseline and time-varying covariates were collected.

### Population PK modeling

Non-linear mixed-effects modeling was performed with NONMEM (version 7.3; ICON Development Solutions, Ellicott City, Maryland, USA) (37) using the FOCE method with interaction and Perl-speaks-NONMEM (version 3.5.3; Uppsala University, Uppsala, Sweden) (38) and R (http://www.R-project.org/).

**Fig 6:**
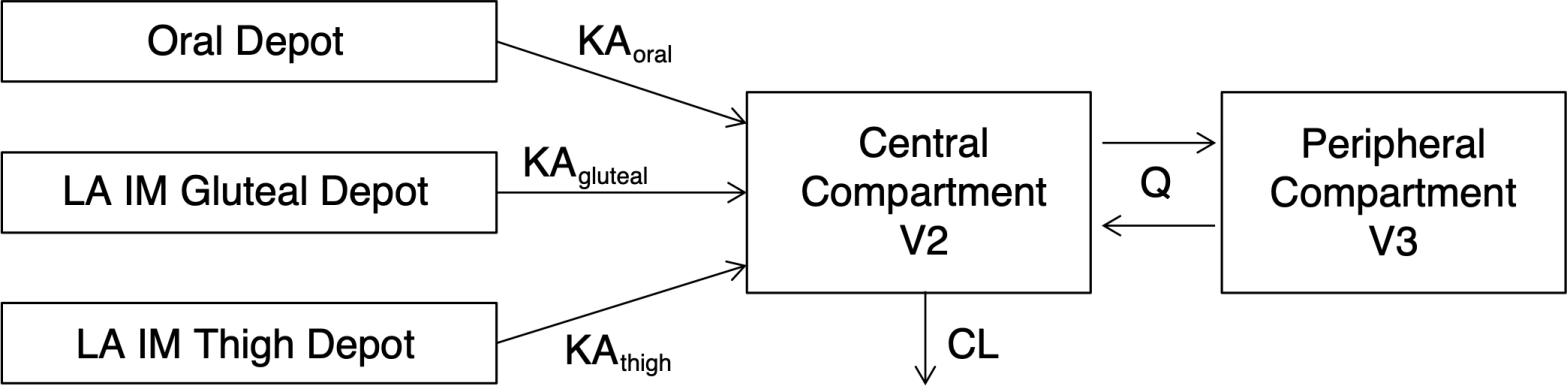
Cabotegravir population pharmacokinetic model with thigh injection. CL, systemic clearance; IM, intramuscular; KA_gluteal_, absorption rate constant for LA IM gluteal injection; KA_oral_, absorption rate constant for the oral tablet; KA_thigh_, absorption rate constant for LA IM thigh injection; LA, long-acting; Q, inter-compartment clearance; V2, central compartment volume of distribution; V3, peripheral compartment volume of distribution.

The oral + gluteal model (16) was a two-compartment model with first-order oral and IM absorption and elimination for both routes of administration. PK parameters included KA_gluteal_ and the absorption rate constant for the oral tablet (KA_oral_), apparent central clearance (CL/F; F represents the unknown absolute bioavailability of CAB LA IM gluteal injection), apparent central volume of distribution (V2/F), apparent inter-compartmental clearance (Q/F), apparent peripheral compartment volume of distribution (V3/F), and *F*_oral/gluteal_. The oral + gluteal model was modified by adding the thigh injection depot compartment with KA_thigh_ and *F*_thigh/gluteal_ and their respective IIV, as shown in Fig. 6. The random effects (ETA) for KA_gluteal_, *F*_oral/gluteal_, KA_thigh_, and *F*_thigh/gluteal_ were estimated separately. The model was fit to PK data following both gluteal and thigh injections, enabling within-person comparison in ATLAS-2M participants who received both gluteal and thigh injections. Concentrations below the LLOQ following LA IM injections were modeled using a likelihood-based M3 method (39–41).

KA_thigh_ and *F*_thigh/gluteal_ were the only parameters related to the thigh injection and, therefore, were estimated. All other parameters were not related to the thigh injection and were, therefore, fixed to the values in the final oral + gluteal model (16). Models were fit to all PK data following oral administration, gluteal injections, and thigh injections from all participants who received at least one thigh injection and had at least one CAB plasma concentration following a thigh injection. While the covariate effects on KA_gluteal_ were fixed (16), the associations of KA_thigh_ and *F*_thigh/gluteal_ with various covariates were estimated using a power function for continuous covariates: $P_{i}=P\times {\left(\frac{COV_{i}}{COV_{median}}\right)}^{\theta_{P}}$, and using fractional change for categorical covariates: $P_{i}=P\times \left(1+\theta_{P}\times {IND}_{i}\right)$, where COV*_i_* is the value of a continuous covariate in the *i*-th participant; IND*_i_* is an indicator of a categorical covariate equal to 0 or 1 in the *i*-th participant; *P_i_* is the typical value of PK parameter *P* in the *i*-th participant (i.e., participants with COV*_i_* or IND_i_); *P* is the typical value of PK parameter *P* in participants with the median value of a continuous covariate (COV_median_) or in the absence of a categorical covariate (IND*_i_* = 0); *θ_P_* is the estimated exponent or fractional change for PK parameter *P*.

KA_thigh_ would be modeled as a function of KA_gluteal_ if both of the following criteria were met: (i) the correlation between KA_gluteal_ and KA_thigh_ was deemed strong with a Pearson correlation coefficient of >0.7 (42–45) and (ii) the covariate relationships on KA_gluteal_ were deemed similar to those on KA_thigh_. Various linear functions and power functions linking KA_thigh_ and KA_gluteal_ would be explored. The statistical criterion for selecting the optimal function was an OFV decrease of 3.84 for increasing 1 degree of freedom (df), i.e., *P* < 0.05. If either of the criteria listed above was not met, the covariate relationships of KA_thigh_ and *F*_thigh/gluteal_ would be evaluated in a stepwise manner by forward addition followed by backward elimination with a significance level of *P* < 0.01 and *P* < 0.001, respectively (OFV decrease of 6.63 and 10.83 for 1 df, respectively).

Bootstrapping (46) and pcVPC (47) were performed using 500 replicates based on the final model. The relative impact of covariates retained in the final model on CAB *C*_tau_-1, *C*_tau_-SS, and *C*_max_-SS concentrations was evaluated through a series of simulations following CAB QM and Q2M IM thigh injections. Both QM- and Q2M-simulated thigh injections started with a CAB IM thigh injection of 600 mg. For QM injections, 400 mg was administered QM after the first injection. For Q2M injections, 600 mg was administered 1 month after the first injection and then Q2M. *C*_tau_-SS and *C*_max_-SS were defined as *C*_tau_ and *C*_max_ following the 11th injection of the QM injections or the 6th injection of the Q2M injections. In each covariate subgroup, 5,000 virtual participants were simulated.

## Data Availability

No new data were created for this analysis. The data that support the findings of this analysis may be available from ViiV Healthcare upon request and approval from www.clinicalstudydatarequest.com. Restrictions apply to the availability of these data.

## SUPPLEMENTAL MATERIAL

Figure S1 aac.00880-24-s0001.tiff Predication-corrected visual predictive check of the final model for (A) study 208832 and (B) ATLAS-2M thigh PK substudy.

Table S1 aac.00880-24-s0002.docx Final model selection.

ASM does not own the copyrights to Supplemental Material that may be linked to, or accessed through, an article. The authors have granted ASM a non-exclusive, world-wide license to publish the Supplemental Material files. Please contact the corresponding author directly for reuse.
