# Supplementary figures and images for: Population pharmacokinetics of cabotegravir following intramuscular thigh injections in adults with and without HIV
Source: Antimicrob Agents Chemother. 2024 Oct 23;68(12):e00880-24. doi: 10.1128/aac.00880-24 (PMC11619381; doi:10.1128/aac.00880-24)

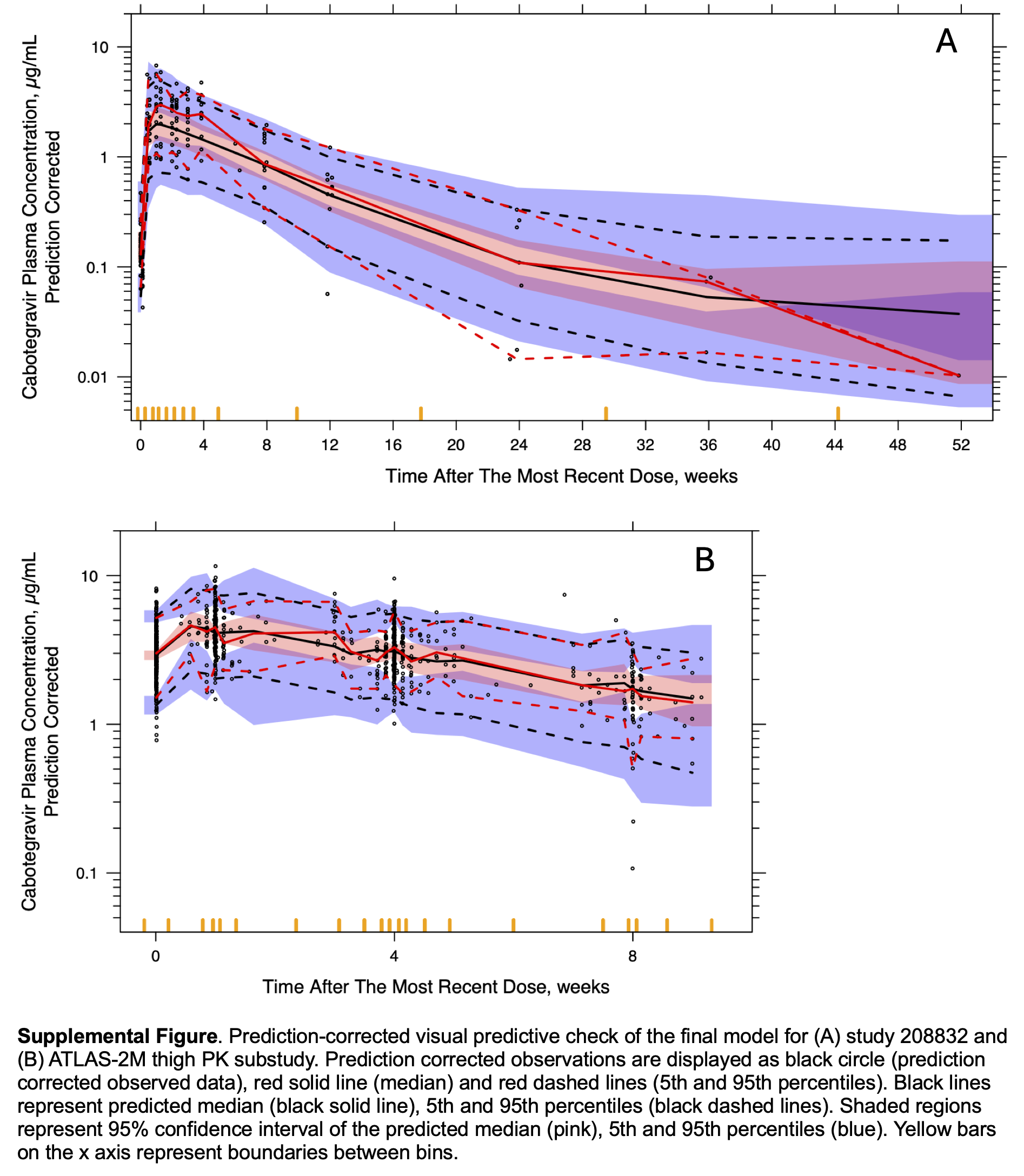

Supplement: Figure S1 — Predication-corrected visual predictive check of the final model for (A) study 208832 and (B) ATLAS-2M thigh PK substudy. [file aac.00880-24-s0001.tiff]
