# Supplementary material for: Population pharmacokinetics of cabotegravir following intramuscular thigh injections in adults with and without HIV
Source: Antimicrob Agents Chemother. 2024 Oct 23;68(12):e00880-24. doi: 10.1128/aac.00880-24 (PMC11619381; doi:10.1128/aac.00880-24)
Supplement: Table S1 — Final model selection. [file aac.00880-24-s0002.docx]

**SUPPLEMENTAL MATERIAL**

**Supplemental Table.** Final model selection.

|  | **Model of fixed effect** | **Model of random effect** | **OFV** | **vs. BASE** | **vs. final model** |
| --- | --- | --- | --- | --- | --- |
| IIV on KA3 | TV_KA3 = TV_KA2 + THETA1 (final model) | KA3 = TV_KA3 * EXP(ETA) | 2229.6 | −29.0 |  |
|  | TV_KA3 = TV_KA2 * THETA1 + THETA2 |  | 2226.8 | −31.8 | -2.8 |
|  | TV_KA3 = THETA1 * (TV_KA2^THETA2) |  | 2226.6 | −32.0 | -3.0 |
| IIV on DKA | TV_DKA = THETA1  KA3 = KA2 + DKA | DKA = TV_DKA * EXP(ETA) | 2242.4 | −16.2 | 12.8 |
|  |  | DKA = TV_DKA + ETA | 2236.2 | −22.4 | 6.6 |
|  |  | DKA = TV_DKA * (1 + ETA) | 2235.7 | −22.9 | 6.0 |
| BASE, modified final oral+gluteal model with no covariate on KA3 and F3 (Figure 6); DKA, additive difference between KA3 and KA2; ETA, random effect; final model, the first model listed in this table; IIV, inter-individual variability; IM, intramuscular; KA2, absorption rate constant for LA IM gluteal injection; KA3, absorption rate constant for LA IM thigh injection; LA, long‑acting; OFV, objective function value; oral+gluteal model, population pharmacokinetic model for oral tablet and gluteal injections; THETA1 and THETA2, fixed effects; TV, typical value. | | | | | |
